# Antibiotic Susceptibility Monitoring of *Neisseria gonorrhoeae* in Bacolod City, Philippines

**DOI:** 10.3390/tropicalmed2030045

**Published:** 2017-08-29

**Authors:** Clark Martin P. Araneta, Alain C. Juayang, Joseph Peter T. Lim, Eleeza Marie G. Quilop, Nadine Joy G. Casaysay, Gene Marie L. Tamesis, Tricia Marie G. Yude, Sarah Joyce E. Romero, Raziel C. Gayoba

**Affiliations:** 1 Medical Technology Program, Colegio San Agustin, Bacolod City 6100, Philippines; alainjuayang@yahoo.com (A.C.J.); nadinejoycasaysay@gmail.com (N.J.G.C.); genemarietamesis@gmail.com (G.M.L.T.); triciayude@yahoo.com (T.M.G.Y.); sweetiepieapplepie@gmail.com (S.J.E.R.); razgayoba2427@gmail.com (R.C.G.); 2 Pathology Department, Dr. Pablo O. Torre Memorial Hospital, Bacolod City 6100, Philippines; djozip@gmail.com (J.P.T.L.); eezaquilop@yahoo.com (E.M.G.Q.)

**Keywords:** antibiotic susceptibility, *Neisseria gonorrhoeae*, Bacolod City

## Abstract

A local study was conducted to monitor the antibiotic susceptibility of *N. gonorrhoeae* in Bacolod City, Philippines. A total of 88 isolates were taken during the period of 1 January 2015 to 30 June 2017, from male patients ages 12 to 72 years. The highest incidence of gonorrhea infection was in the group aged 20–24 years (34.09%). The susceptibility pattern to antibiotics was as follows: ceftriaxone 100%, cefixime 82.6%, spectinomycin 92.1%, ciprofloxacin 4.9%, tetracycline 5.1%, and penicillin G with 0%. All isolates were noted to produce beta-lactamase, which can be attributed to plasmid-mediated penicillin resistance. These findings indicate that the resistance rates of *N. gonorrhoeae* to most commonly-used antibiotics are increasing, and that ceftriaxone remains an effective antibiotic in treating gonorrhea infections locally.

## 1. Introduction

*Neisseria gonorrhoeae* is a Gram-negative diplococcus that causes gonorrhea, a common communicable disease that is spread from person to person through sexual contact. Gonorrhea is considered to be endemic worldwide and causes clinical manifestations in infected individuals such as urethritis, cervicitis, pharyngitis, proctitis, conjunctivitis, disseminated blood stream infection, tubal infection with impaired infertility, and the spread of infection to the prostate and epididymis [1]. Pediatric patients are not exempted from gonococcal infections. Neonates of infected mothers that are delivered by normal spontaneous vaginal delivery are prone to gonococcal conjunctivitis [2]. 

Although gonorrhea is considered an old malady that can be traced back to ancient times, this type of sexually transmitted infection has persisted through the years [3]. As of 2008, World Health Organization (WHO) estimated the emergence of approximately 106 million new cases per year [4]. In addition, there are a number of documented studies stating that *N. gonorrhoeae* has become resistant to most or even to all antibiotics commonly used to treat it. Reports state that *N. gonorrhoeae* has exhibited decreased susceptibility or non-susceptibility to front-line extended spectrum cephalosporins (cefixime and ceftriaxone) [5,6,7,8] and azithromycin [7,9,10,11]. Resistance to these extended spectrum cephalosporins has already been verified in several countries including Japan, Australia, Canada, Europe, South Africa, and others [3], and the organism has been classified by the Centers for Disease Control and Prevention (CDC) as a ‘superbug’ [3] that is difficult, if not at times impossible, to treat [12]. 

Data regarding the resistance of *N. gonorrhoeae* are commonly available on the World Wide Web, be it worldwide [12] or countrywide [13]. A local five-year study by Juayang et al. [14] that was published in 2015 reported that cefixime and ceftriaxone, having a 100% susceptibility rate, were still noted to be effective against *N. gonorrhoeae*. Recently, a number of news articles have been circulating about this superbug or untreatable gonorrhea [12]. Thus, to provide epidemiological markers for laboratory surveillance of *N. gonorrhoeae*, this continuing local study was undertaken.

## 2. Materials and Methods

### 2.1. Inclusion and Exclusion Criteria

This study focuses on male patients of Dr. Pablo O. Torre Memorial Hospital that were sent to the laboratory for the culture of their urethral discharges for *N. gonorrhoeae* between 1 January 2015 and 30 June 2017. Data from these patients were then grouped according to the origin of the sample, the age groups, and the isolates’ antimicrobial test results.

### 2.2. Bacterial Isolation and Identification

Samples were collected by trained personnel from patients suspected to have gonococcal infections, using a sterile cotton swab. Swabs were inoculated onto chocolate agar plates (Oxoid) and incubated at 37 °C for 24 h with 5–8% carbon dioxide and a humidified atmosphere. Positive cultures were observed with characteristics of small, grayish colonies. Suspected colonies were then inoculated onto another chocolate agar plate and incubated at 37 °C for 24 h in a carbon dioxide incubator. Purified colonies were then tested biochemically using superoxol, oxidase, Gram stain, sugar fermentation [15], and resistance to colistin [16]. Isolates that were observed to be Gram-negative diplococci, superoxol- and oxidase-positive, able to ferment only glucose, and resistant to colistin, were considered to be *N. gonorrhoeae*.

### 2.3. Antibiotic Susceptibility Testing

Testing for the isolates’ susceptibility was carried out by Kirby-Bauer disc diffusion assay [15,17] in accordance with the guidelines and criteria of the Clinical Laboratory Standards Institute [18] and the Centers for Disease Control and Prevention [19].

Three to five colonies from an overnight culture were suspended in normal saline solution adjusted to 0.5 McFarland standard and mixed vigorously to break up clumps. A sterile applicator stick was moistened in a standardized suspension, and excess moisture was expressed by rotating the swab against the tube wall. The entire surface of the GC agar (Oxoid) supplemented with 1% vitox (Oxoid) was then inoculated in three different directions to ensure uniform and confluent growth. The plates were allowed to stand for three to five minutes before applying the antibiotic discs. Commonly-used antibiotics (Oxoid) including cefixime (30 µg), ceftriaxone (30 µg), ciprofloxacin (5 µg), penicillin G (10 units), spectinomycin (100 µg), and tetracycline (30 µg) were applied two to three centimeters away from the edge of the plate and each other. Azithromycin was not included for antibiotic testing because the breakpoints for the interpretation of its zone of inhibition using the Clinical and Laboratory Standards Institute (CLSI) [18] guidelines are not available.

The plates for susceptibility testing were then incubated in inverted positions at 37 °C for 20 to 24 h in a carbon dioxide incubator. After 20 to 24 h, plates were retrieved, and the zones of inhibition were measured using a vernier caliper. Breakpoints for the definition of susceptibility were defined as zones of inhibition that were equal to or more than 35 mm for ceftriaxone, 31 mm for cefixime, 18 mm for spectinomycin, 38 mm for tetracycline, 41 mm for ciprofloxacin, and 47 mm for penicillin G [18]. Resistance was defined as having zones of inhibition that were equal to or less than 14 mm for spectinomycin, 30 mm for tetracycline, 27 mm for ciprofloxacin, and 26 mm for penicillin G [18]. 

### 2.4. Beta-Lactamase Production

Beta-lactamase production was determined using a cefinase (nitrocefin-based) disc [14,16,18]. This was done by placing a cefinase disc on a glass slide and moistening with a small drop of deionized water. A colony from the chocolate agar was then taken and smeared on the cefinase disc. A change of color from yellow to pink indicated beta-lactamase production. 

### 2.5. Quality Control

The quality of all culture media, biochemical tests, and potency of antibiotics were tested using the reference strain *N. gonorrhoeae* ATCC 49226 at regular intervals. 

### 2.6. Data Analysis

Data entry and analysis of the frequency of variables were made using the WHONET version 5.6 software downloaded from the WHO website. 

### 2.7. Ethical Consideration

Ethics approval was obtained from Dr. Pablo O. Torre Memorial Hospital’s Research Ethics and Review Committee, approval number RERC 2016-17.

### 2.8. Results

A total of 88 *N. gonorrhoeae* isolates coming from 88 male patients were collected between 1 January 2015 and 30 June 2017. Among these patients, nine (10.23%) were considered pediatric (18 years old or younger) and 79 (89.77%) were considered adults. 

Moreover, results showed that a 12-year-old was the youngest and a 72-year-old was the oldest patient from whom the organisms were isolated (Table S1 and Figure S1). Gonococcal infections were also noted to be the most prevalent in the group aged 20–24 years (30, 34.09%) followed by the group aged 25–29 years (15, 17.05%). Frequency of *N. gonorrhoeae* by age group is shown in Table 1.

Ceftriaxone remains the most effective drug with a susceptibility rate of 100%, followed by spectinomycin and cefixime with 92.1% and 80.9%, respectively. Penicillin G was noted to be the most ineffective with 0% susceptibility, followed by ciprofloxacin and tetracycline with 4.9% and 5.1%, respectively. The summary of the percent susceptibility of *N. gonorrhoeae* is shown in Table 2.

All 88 (100%) isolates were beta-lactamase producers, and 33 out of 78 (42.31%) that were tested with tetracycline were noted to have zones of inhibition that were below 19 mm (Table S1), usually indicating a plasmid-mediated tetracycline-resistant *N. gonorrhoeae*. 

## 3. Discussion

*N. gonorrhoeae* poses a health problem by developing resistance to commonly-used antibiotics, not just in developing countries but worldwide. It infects individuals regardless of age group or sexual orientation; thus, periodic monitoring is essential for the early detection of drug resistance.

In this study, the youngest patient was noted to be 12 years old, and the pathogen is most frequently found in the group aged 20–24 years. This can be probably attributed to the lifestyle and the laws that the Philippines is currently adopting. As stated in the Anti-Rape Law of 1997 (RA 8353), the age for consensual sex in the Philippines is 12. Recently, the Department of Health, Philippines, issued a statement that millennials are mostly affected by sexually transmitted disease, especially HIV [20]. This age group also coincides with the data of Golparian et al. [21], Ali et al. [15], as well as the previous study of Juayang et al. [14].

Though a lot of recent reports have circulated the idea of the superbug, ceftriaxone remains the most effective drug in this study, wherein all isolates were found to be susceptible, since that reported in the study of Juayang et al. [14] in 2015. This finding is similar to the study of Ali et al. [15] in Ethiopia, Toliman et al. [16] in Papua New Guinea, and Hsueh et al. [17] in northern Taiwan. 

In this study, a total of 15 (17.4%) isolates were found to be non-susceptible to cefixime. Though the method utilized was disc diffusion, this can still be used to identify the susceptibility of *N. gonorrhoeae* to antimicrobial agents [18,19] and is at par with E-tests and agar dilution tests [16,17]. Cefixime at any dosage is no longer recommended by the CDC as a first-line regimen for gonococcal infection [22]. Cefixime is only considered as an alternative to an effective cephalosporin regimen because it cannot provide high or sustainable bactericidal levels such as those of ceftriaxone [23]. In the event that cefixime is used as an alternative regimen, the CDC [22,23] recommends that the patient should return after a week for a test-of-cure at the site of infection. As of 7 July 2017, the updated global treatment recommendations of World Health Organization advise doctors to give two antibiotics, namely, ceftriaxone and azithromycin [12]. Non-susceptibility to cefixime [12] has already been noted in numerous studies such as those of Kovari et al. [24] in Switzerland, and Cole et al. [25] in Europe.

Resistance to tetracycline and ciprofloxacin in Bacolod City, Philippines has increased to 74.4% and 78%, respectively, when compared to the study of Juayang et al. [14] in 2015 (69% and 75.3%). High levels of resistance to these antibiotics were also observed in northern Taiwan [17], Hong Kong [26], Switzerland [24], and Ethiopia [15]. In the Philippines, good susceptibility to fluoroquinolones was noted in the early 90s [27]; however, resistance to fluoroquinolones and tetracyclines in the country was already documented in the mid-90s [28]. In 2007, the emergence of fluoroquinolone resistance in the United States prompted the CDC to no longer recommend this antibiotic for the treatment of gonorrhea [22].

Penicillin G resistance, observed to be 88%, was attributed to the ability of the organism to produce beta-lactamase, as also seen in the previous study of Juayang et al. [14]. Beta-lactamase production capability of the isolates provides epidemiological information and recognition of plasmid-mediated penicillin resistance [17]. Although penicillin G is not currently used in the treatment of gonorrhea, it is still advisable to monitor susceptibility, because it is used in other parts of the globe and prevalence of resistance remains high [15]. 

Spectinomycin resistance of 6.7% was lower from that found by Juayang et al. [14], which was 8%. Spectinomycin is used as an alternative in cases where patients are allergic to beta-lactam drugs. However, spectinomycin cannot effectively eradicate pharyngeal gonorrhea because of its poor excretion in the saliva [29]. Although the resistance rate is low, the WHO does not recommend the use against gonorrhea of antibiotics that have resistance rates of more than 5% [12,21]. 

Resistance varies with time and with regions or countries, and it can emerge rapidly in any locality. Antibiotic resistance patterns in a locality can change due to the importation of new strains or the development of resistance. Throughout the world, the antibiotic resistance of *N. gonorrhoeae* continues to rise through the years beginning with penicillin, tetracycline, fluoroquinolones, and recently the extended spectrum cephalosporins [13]. Resistance is acquired in different ways. The primary causes, however, are overuse, inappropriate drug prescription, extensive agricultural use, availability of few antibiotics, and regulatory barriers [30]. A typical scenario for most organisms is to adapt and evolve every time a new class of antibiotics is discovered and used. Countrywide, the data of Antimicrobial Resistance Surveillance Program (ARSP) [13] indicates that both cefixime and ceftriaxone can be used to treat gonococcal infection. Unfortunately, as observed in this study, only ceftriaxone remains the drug of choice for treatment of gonorrhea in the locality of Bacolod City, Philippines. To ensure effective treatment, continuous surveillance of antibiotic resistance is imperative and must be population-based [21]. 

## 4. Conclusions

Finally, the prevalence of high resistance rates of *N. gonorrhoeae* against ciprofloxacin, tetracycline, and penicillin G was observed. Non-susceptibility to cefixime was also noted, and decreasing resistance in spectinomycin was observed. The results of this study also conform to the data and recommendation of the CDC and the WHO for the use of ceftriaxone as the first-line regimen against gonorrhea in Bacolod City, Philippines. Continued monitoring of the resistance patterns of *N. gonorrhoeae* is recommended in order to detect early resistance to ceftriaxone.

## Figures and Tables

**Table 1:** Frequency of isolated *N. gonorrhoeae* from male patients by age group.

Age Group (Years)	Frequency
10–14	2 (2.27%)
15–19	8 (9.09%)
20–24	30 (34.09%)
25–29	15 (17.05%)
30–34	13 (14.77%)
35–39	5 (5.68%)
40–44	4 (4.55%)
45–49	0 (0%)
50–54	2 (2.27%)
55–59	2 (2.27%)
≥60	7 (7.95%)

**Table 2:** Drug resistance pattern of *N. gonorrhoeae* in Bacolod City, Philippines.

Antibiotics	No. of Isolates Tested	Susceptible	Non-Susceptible	Intermediate	Resistant
Ceftriaxone	88	100% (88/88)	0% (0/88)	-	-
Cefixime	86	82.6% (71/86)	17.4% (15/86)	-	-
Spectinomycin	86	91.8% (79/86)	-	1.2% (1/86)	7.0% (6/86)
Tetracycline	78	5.1% (4/78)	-	20.5% (16/78)	74.4% (58/78)
Ciprofloxacin	82	4.9% (4/78)	-	17.1% (14/82)	78% (64/82)
Penicillin G	88	0% (0/88)	-	0% (0/88)	100% (88/88)

## Supplementary Materials

Available online at http://www.mdpi.com/2414-6366/2/3/45/s1.

## References

[1] Crowley L. (2007). Communicable Diseases in an Introduction to Human Disease: Pathology and Pathophysiology Correlations.

[2] Hammerschlag M., Sleiman J., Shah S. (2009). Conjunctivitis in the Neonate in Pediatric Practice Infectious Disease.

[3] Unemo M., del Rio C., Shafer W. (2016). Antimicrobial resistance expressed by *Neisseria gonorrhoeae*: A major global public health problem in the 21st century. Microbiol. Spectr..

[4] Ndowa F., Lusti-Narasimhan M. (2012). The threat of untreatable gonorrhoea: Implications and consequences for reproductive and sexual morbidity. Reprod. Health Matters.

[5] Allen V., Mitterni L., Seah C., Rebbapragada A., Martin I., Lee C., Siebert H., Towns L., Melano R., Low D. (2013). *Neisseria gonorrhoeae* treatment failure and susceptibility to cefixime in Toronto, Canada. JAMA.

[6] Lahra M., Enriquez R. (2016). Australian gonococcal surveillance programme, 1 April to 30 June 2016. Commun. Dis. Intell. Q. Rep..

[7] Wind C.M., Schim van der Loeff M.F., van Dam A.P., de Vries H.J., van der Helm J.J. (2017). Trends in antimicrobial susceptibility for azithromycin and ceftriaxone in *Neisseria gonorrhoeae* isolates in Amsterdam, the Netherlands, between 2012 to 2015. Eurosurveillance.

[8] Gong Z., Lai W., Liu M., Hua Z., Sun Y., Xu Q., Xia Y., Zhao Y., Xie X. (2016). Novel genes related to ceftriaxone resistance found among ceftriaxone-resistant *Neisseria gonorrhoeae* strains selected in vitro. Antimicrob. Agents Chemother..

[9] Regnath T., Mertes T., Ignatius R. (2016). Antimicrobial resistance of *Neisseria gonorrhoeae* isolates in south-west Germany, 2004 to 2015: Increasing minimal inhibitory concentrations of tetracycline, but no resistance to third-generation cephalosporins. Eurosurveillance.

[10] Brunner A., Nemes-Nikodem E., Jeney C., Szabo D., Marschalko M., Karpati S., Ostorhazi E. (2016). Emerging azithromycin resistance among the *Neisseria gonorrhoeae* strains isolated in Hungary. Ann. Clin. Microbiol. Antimicrob..

[11] Yasuda M., Hatazaki K., Ito S., Kitanohara M., Yoh M., Kojima M., Narita H., Kido A., Miyata K., Dequch T. (2017). Antimicrobial susceptibility of *Neisseria gonorrhoeae* in Japan from 2000 to 2015. Sex. Transm. Dis..

[12] World Health Organization Antibiotic resistance gonorrhoea on the rise, new drugs needed. http://www.who.int/mediacentre/news/releases/2017/Antibiotic-resistant-gonorrhoea/en/.

[13] (2016). Antibiotic Resistance Surveillance Program.

[14] Juayang A., Lim J.P., Acosido M.A., Maestral D., Gallega C. (2015). Antibiotic susceptibility of *Neisseria gonorrhoeae* in Bacolod City, Philippines. BMRJ.

[15] Ali S., Sewunet T., Sahlemariam Z., Kibru G. (2016). *Neisseria gonorrhoeae* among suspects of sexually transmitted infection in Gambella Hospital, Ethiopia: Risk factors and drug resistance. BMC Res. Notes.

[16] Toliman P., Lupiwa T., Law G., Reeder J., Siba P. (2010). *Neisseria gonorrhoeae* isolates from four centres in Papua New Guinea remain susceptible to amoxycillin-clavulanate therapy. P. N. G. Med. J..

[17] Hsueh P.R., Tseng S.P., Teng L.J., Ho S.W. (2005). High prevalence of ciprofloxacin resistant *Neisseria gonorrhoeae* in Northern Taiwan. Clin. Infect. Dis..

[18] Clinical Laboratory Standards Institute (2016). Performance Standards for Antimicrobial Susceptibility Testing.

[19] Centers for Disease Control and Prevention *Neisseria gonorrhoeae* reference strains for antimicrobial susceptibility testing. https://www.cdc.gov/std/gonorrhea/arg/b88-feb-2005.pdf.

[20] Department of Health Millenials. na HIV Positive Dumami. (Millenials that are HIV positive are increasing in numbers).

[21] Golparian D.B.T. (2014). First antimicrobial resistance data and genetic characteristics of *Neisseria gonorrhoeae* isolates from Estonia, 2009–2013. New Microbes New Infect..

[22] Centers for Disease Control and Prevention (2012). Update to CDC’s sexually transmitted diseases treatment guidelines, 2010: Oral cephalosporins no longer recommended treatment for gonococcal infections. Morb. Mortal. Wkly. Rep. (MMWR).

[23] Centers for Disease Control and Prevention (2015). Sexually transmitted disease treatment guidelines, 2015. Morb. Mortal. Wkly. Rep. (MMWR).

[24] Kovari H., de Melo O., Hauser M., Lauchli S., Meyer J., Weber R., Zbinden R. (2013). Decreased susceptibility of *Neisseria gonorrhoeae* isolates from Switzerland to cefixime and ceftriaxone: Antimicrobial susceptibility data from 1990 and 2000 to 2012. BMC Infect. Dis..

[25] Cole M., Spiteri G., Town K., Unemo M., Hoffmann S., Chisholm S., Amato-Gauci A., van de Laar M., Ison C. (2014). Risk factors for antimicrobial-resistant *Neisseria gonorrhoeae* in Europe. Sex. Transm. Dis..

[26] Lo J.Y.C., Man Ho K., Chun T., Lo A. (2012). Surveillance of gonococcal antimicrobial susceptibility resulting in early detection of emerging resistance. J. Antimicrob. Chemother..

[27] Clendennen T.E., Hames C.S., Kees E.S., Price C., Rueppel W.J., Andrada A.B., Espinosa G.E., Kabrerra G., Wignall F.S. (1992). In vitro antibiotic susceptibilities of *Neisseria gonorrhoeae* isolates in the Philippines. Antimicrob. Agents Chemother..

[28] de los Reyes M.R., Pato-Mesola V., Klausner J., Manalastas R., Wi T., Tuazon C., Dallabetta G., Whittington W., Holmes K. (2001). A randomized trial of ciprofloxacin versus cefixime for treatment of gonorrhea after rapid emergence of gonococcal ciprofloxacin resistance in The Philippines. Clin. Infect. Dis..

[29] Gil-Setas A., Navascues-Ortega A., Beristain X. (2010). Spectinomycin in the treatment of gonorrhoea. Euro Surveill..

[30] Lee Ventola C. (2015). The antibiotic resistance crisis. Part 1: Causes and threats. Pharm. Ther..

