# Supplementary material for: Antibiotic Susceptibility Monitoring of Neisseria gonorrhoeae in Bacolod City, Philippines
Source: Trop Med Infect Dis. 2017 Aug 29;2(3):45. doi: 10.3390/tropicalmed2030045 (PMC6082094; doi:10.3390/tropicalmed2030045)

Supplementary Materials: Antibiotic Susceptibility Monitoring of *Neisseria gonorrhoeae* in Bacolod City, Philippines

Supplementary Table 1. Isolate Listing of the *N. gonorrhoeae* Isolated from Urethral Discharges of Men from Bacolod City, Philippines with Date Range January 01, 2016 to June 30, 2017.

ID Number	Age	Location Type	Specimen Date (DD/MM/YYYY)	*Beta*-Lactamase	Ceftriaxone	Cefixime	Penicillin G	Ciprofloxacin	Spectinomycin	Tetracycline
WA 0156-17	36	out	8/2/2017	Positive	40	36	6	25	27	31
WA 0504-17	38	out	24/05/2017	Positive	37	37	6	24	28	30
WA 0590-17	61	out	17/06/2017	Positive	39	38	6	18	22	12
WA-0273-17	22	out	14/03/2017	Positive	39	40	6	15	22	15
WA0021-15	20	out	1/4/2016	Positive	35	31	13	43	24	
WA0030-15	25	out	1/8/2015	Positive	42	38	24	34	30	38
WA0036-16	27	out	1/9/2016	Positive	37	33	6	14	23	14
WA0072-16	22	out	18/01/2016	Positive	36	31	6	18	26	31
WA0074-16	21	out	18/01/2016	Positive	37	24	6	32	20	25
WA0083-16	43	out	23/01/2017	Positive	40	39	6	20	21	14
WA0089-16	24	out	25/01/2016	Positive	35	33	18	37	30	17
WA0122-15	25	out	15/02/2015	Positive	40	42	9	17	32	12
WA0125-17	26	out	1/2/2017	Positive	38	38	6	35	25	36
WA0135-15	34	out	18/02/2015	Positive	36	33	6	16	28	35
WA0141-16	18	out	2/9/2016	Positive	43	42	6	15	25	16
WA0161-15	26	out	23/01/2015	Positive	39	35	13	24	27	
WA0168-16	21	out	17/02/2016	Positive	40	44	6	19	25	16
WA0172-15	20	out	27/02/2015	Positive	46	37	6	24	35	19
WA0181-17	60	out	14/02/2017	Positive	39	39	6	21	24	27
WA0184-16	30	out	20/02/2016	Positive	36	37	6	24	14	35
WA0188-16	33	out	23/02/2016	Positive	39	38	6	20	24	13
WA0202-15	25	out	29/02/2016	Positive	44	42	6	24	30	20
WA0204-16	22	out	3/1/2016	Positive	36	38	6	8	25	15
WA0205-16	51	out	3/1/2016	Positive	35	40	6	23	27	20
WA0214-16	20	out	3/3/2016	Positive	36	33	6	13	20	10
WA0223-17	67	out	28/02/2017	Positive	39	38	6	15	22	6
WA0230-17	30	out	2/3/2017	Positive	39	39	6	19	18	15
WA0282-15	17	out	4/7/2015	Positive	41	40	22	32	34	34
WA0293-16	20	out	25/03/2016	Positive	37	38	6	21	20	15
WA0294-15	21	out	4/8/2015	Positive	38	44	6	35	25	32
WA0323-17	38	out	31/03/2017	Positive	38	20	10	17	11	14

WA0326-15	21	out	17/04/2015	Positive	38	33	14	25	32	39
WA0331-17	20	out	3/4/2017	Positive	36		6	6		30
WA0350-17	72	out	18/04/2017	Positive	39	39	6	25	35	6
WA0353-17	17	out	11/4/2017	Positive	42	19	6	25	36	37
WA0385-15	27	out	5/8/2015	Positive	36	33	6	31	28	6
WA0391-16	41	out	26/04/2016	Positive	35	6	6	27	24	16
WA0400-17	30	out	21/04/2017	Positive	42	39	6	18	26	10
WA0406-16	17	out	30/04/2016	Positive	48	42	6	17	35	31
WA0423-17	19	out	8/5/2017	Positive	44	36	6	25	25	32
WA0424-17	53	out	8/5/2017	Positive	35	38	7	21	20	21
WA0466-15	16	out	1/6/2015	Positive	46	35	6		22	28
WA0472-17	27	out	17/05/2017	Positive	39	39	6	15	20	13
WA0482-17	22	out	20/05/2017	Positive	39	39	6	17	28	10
WA0483-15	22	out	8/6/2015	Positive	44	37	15		29	20
WA0496-15	31	out	11/6/2015	Positive	38	35	12		37	28
WA0512-16	33	out	2/6/2016	Positive	41	26	6	15	24	24
WA0526-17	28	out	30/05/2017	Positive	39	38	6	18	25	28
WA0528-16	26	out	8/6/2016	Positive	40	6	6	6	8	12
WA0532-16	17	out	9/6/2016	Positive	42	41	6	18	25	32
WA0550-16	20	out	15/06/2016	Positive	41	38	6	22	23	26
WA0558-17	27	out	6/6/2017	Positive	39	32	6	6	22	12
WA0560-17	66	out	8/6/2017	Positive	37	37	6	17	19	28
WA0573-16	22	out	21/06/2016	Positive	41	38	6	22	23	26
WA0593-16	24	out	29/06/2016	Positive	38	35	11	35	30	31
WA0595-16	24	out	29/06/2016	Positive	38	35	10	35	30	31
WA0599-15	23	out	17/07/2015	Positive	35	32	6	12	23	11
WA0600-17	12	out	22/06/2017	Positive	38	10	6	12	28	32
WA0601-16	37	out	1/7/2016	Positive	39	29	6	12	19	28
WA0615-17	34	out	22/06/2017	Positive	39			18		24
WA0621-16	57	out	7/1/2016	Positive		29	6		20	28
WA0627-17	29	out	7/3/2017	Positive	35	32		39	25	27
WA0629-16	34	out	7/3/2017	Positive	35	27	6		25	12
WA0639-15	33	out	1/8/2015	Positive	37	47	14	17	30	38
WA0653-16	22	out	22/07/2016	Positive	39	40	6	18	27	15
WA0702-16	40	out	2/8/2016	Positive	42	40	6	20	28	16
WA0716-16	70	out	8/5/2016	Positive	39	34	6	20	9	29
WA0764-15	24	out	16/09/2015	Positive	37	26	6	29	20	38
WA0786-15	34	out	23/09/2015	Positive	36	36	13	22	29	30
WA0828-16	30	out	19/09/2016	Positive	40	20	9	22	21	16
WA0830-16	26	out	20/09/2016	Positive	41	22	15	36	11	6
WA0837-16	21	out	20/09/2016	Positive	38	6	6	19	22	15

WA0844-16	27	out	24/09/2016	Positive	42	39		46	27	31
WA0859-16	23	out	5/11/2015	Positive	44	42	6	32	30	
WA0874-15	23	out	5/11/2015	Positive	35	34		13	20	
WA0889-15	14	out	10/11/2015	Positive	35	33	6	18	20	
WA0922-15	30	out	20/11/2015	Positive	35	33	6	10	23	
WA0942-15	58	out	28/11/2015	Positive	35	32	6	14	22	
WA0954-16	43	out	31/10/2016	Positive	38	6	6	14	17	11
WA0969-15	56	out	5/12/2015	Positive	45	34	6	11	23	
WA1014-15	21	out	21/12/2015	Positive	36	33	6	20	24	
WA1024-15	22	out	25/12/2015	Positive	41	34	6	21	6	
WA1050-16	21	out	28/11/2016	Positive	43	35		16	25	32
WA1086-16	42	out	12/9/2016	Positive	43	31	10	20	24	30
WA1113-16	35	out	21/12/2016	Positive	36	37	6	43	23	27
WA1118-16	22	out	22/12/2016	Positive	39	31		38	25	28
WA1123-16	18	out	26/12/2016	Positive	43	43	17		24	18
WA5388-16	65	out	29/12/2016	Positive	44	44	18	43	28	20

Supplementary Figure 1: Age of *Neisseria gonorrhoeae* Infected Patients in Bacolod City, Philippines from January 2015 to June 30, 2017

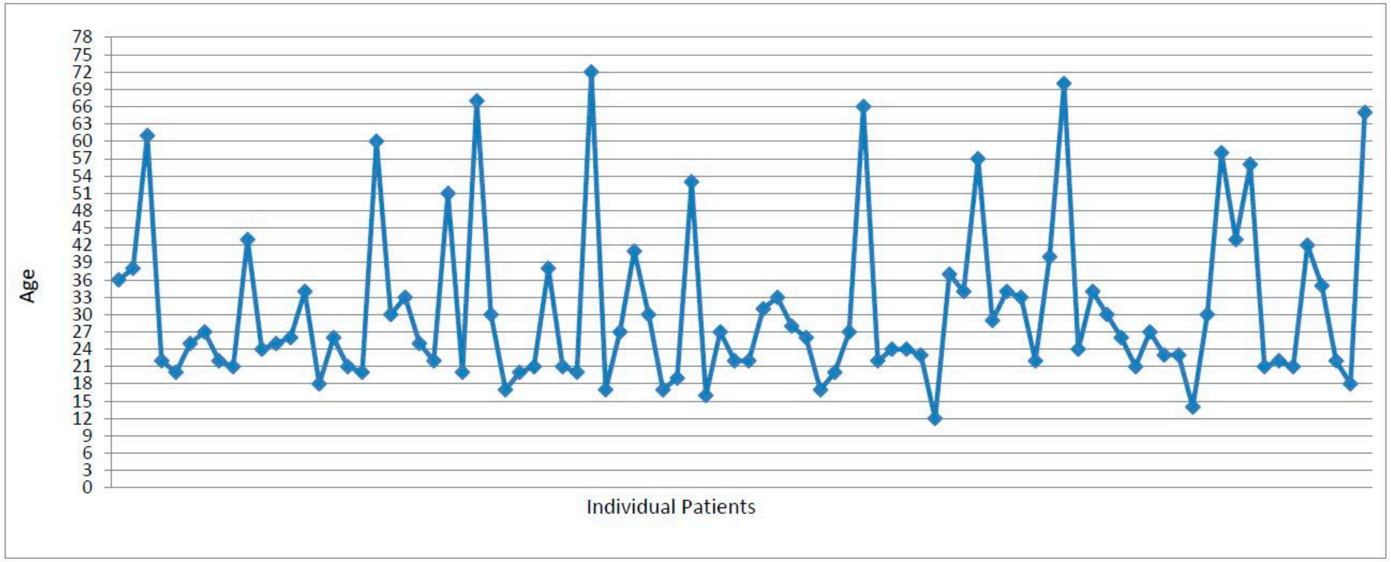

Supplement: Supplementary file 1 [file tropicalmed-02-00045-s001.pdf]
